# The Bitter Truth about Morality: Virtue, Not Vice, Makes a Bland Beverage Taste Nice

**DOI:** 10.1371/journal.pone.0041159

**Published:** 2012-07-18

**Authors:** Kendall J. Eskine, Natalie A. Kacinik, Gregory D. Webster

**Affiliations:** 1 Department of Psychological Sciences, Loyola University, New Orleans, Louisiana, United States of America; 2 Department of Psychology, The Graduate Center and Brooklyn College of the City University of New York, New York, New York, United States of America; 3 Department of Psychology, University of Florida, Gainesville, Florida, United States of America; Royal Holloway, University of London, United Kingdom

## Abstract

To demonstrate that sensory and emotional states play an important role in moral processing, previous research has induced physical disgust in various sensory modalities (visual, tactile, gustatory, and olfactory modalities, among others) and measured its effects on moral judgment. To further assess the strength of the connection between embodied states and morality, we investigated whether the directionality of the effect could be reversed by exposing participants to different types of moral events prior to rating the same neutral tasting beverage. As expected, reading about moral transgressions, moral virtues, or control events resulted in inducing gustatory disgust, delight, or neutral taste experiences, respectively. Results are discussed in terms of the relation between embodied cognition and processing abstract conceptual representations.

## Introduction

Recent years have seen a proliferation of research investigating the relationship between morality and one’s perceptual and emotional states. Since the influential proposal of *social intuitionism* theory [1], numerous studies have demonstrated that emotions play a guiding role in determining moral judgment [2], [3] and that embodied experiences in particular can be a powerful source of information when making moral judgments.

Morality has been conceptualized in terms of a *purity metaphor* [4], [5], [6], and a salient set of recent findings involving physical and moral disgust has provided support for that type of conceptualization [7], [8], [9], [10], [11], [12], [13]. For example, drawing from the common moral vernacular of expressions like “dirty hands” and “dirty mouths” researchers tested whether the moral-purity metaphor was in fact modality-specific [14]. Participants completed a role-playing scenario during which they committed a moral transgression (a lie) either via email (with their hands) or voicemail (with their mouths) prior to engaging in a seemingly unrelated consumer research task where they rated various products such as hand-sanitizer and mouthwash. Participants who lied with their mouths indicated a stronger preference for the mouthwash than other products, whereas participants who lied with their hands showed a stronger preference for hand-sanitizer. These results suggest that people feel a need to physically “purify” themselves after committing a moral transgression.

However, the status of emotions in moral processing is still a subject of lively debate. Some argue that disgust is not really a moral emotion [15], while others question whether moral disgust really qualifies as emotional disgust [16]. The present research cannot provide full or complete answers to these questions, but it was designed to further investigate and test the robustness of the connection between physical and moral disgust by extending the effects reported in other research [9]. Participants in this study were given a disgusting, sweet, or control beverage to drink before making moral judgments. Those who consumed the disgusting, bitter beverage (Swedish Bitters) made significantly harsher moral judgments than those who consumed non-disgusting beverages (Minute Maid Berry Punch or water).

The present research was aimed at extending these findings in a couple of ways. One goal was to show that the relationship between morality and gustation is bidirectional, specifically that processing different moral events can also differentially affect taste perception. This is theoretically important because the embodiment of abstract concepts is often framed in terms of conceptual metaphor theory [17], [18]. This theory posits that embodied information (like taste) provides the “raw material” (i.e., information) that makes up one’s source domain which is subsequently mapped onto an abstract target domain like morality. The relationship between source and target domains was originally proposed to be unidirectional, such that embodied and sensorimotor states could influence the representation and processing of abstract concepts but not vice versa. However, as recently noted elsewhere [19], some studies have shown that “abstract” domains can influence bodily states [20]. Those studies tell us more about conceptual processing than representation, but it is reasonable to extend this logic and argue that source and target domains resulting in bidirectional effects are more likely to be part of the same conceptual representation than domains that are simply unidirectional.

To our knowledge, no research to date has demonstrated that physical and moral disgust are bidirectional. A few clever studies have shown moral purity effects that *imply* disgust [14], [21], [5], [6], but since both physical and moral disgust were not *explicitly* measured, the proposed bidirectionality of these effects remains an open question.

Another goal of the present research was to significantly extend knowledge on morality and embodied cognition to include moral virtue. Hume argued that moral judgments are formed on the basis of both negatively *and* positively valenced emotions [22]. On his view, we judge moral events to be wrong when they make us *feel* bad and virtuous when they make us *feel* good. Theoretically, it follows that instantiating positive emotional arousal in people should positively bias people’s moral processing. There is little research on the relationship between moral virtue and embodiment, although other researchers have used the moral purity metaphor to study the effects of clean scents on prosocial behavior [4]. To further explore and understand the underlying conceptual processes that link embodied states to morality, it is important to demonstrate contrast effects in physical and moral delight in addition to physical and moral disgust. Virtue is as critical to morality as vice, and while there is an abundance of research involving the latter, the former is empirically impoverished from the embodiment perspective. However, to have a complete understanding of the relationship between morality and embodiment–and the extent to which these domains overlap–research must examine a *variety* of moral event types and their differential emotional states.

To test the reversibility of previous findings [9] about the link between gustatory disgust and moral judgments, participants were primed by reading about morally wrong, virtuous, or neutral events prior to rating the same neutral-tasting beverage. It was predicted that exposure to moral transgressions would induce gustatory disgust, whereas reading about morally virtuous behavior would induce gustatory delight.

## Methods

### Participants and Procedure

Sixty (29 females) undergraduates were recruited from the Brooklyn College Psychology participant pool. They were told that the present experiment was a two-part study; part one explored moral judgments, whereas part two involved a taste-perception survey as part of a collaboration with a market research/consumer interest company.

#### Moral vignette manipulation

Groups of 20 participants were randomly assigned to read two vignettes depicting moral transgressions, moral virtues, or control scenarios in a between-subjects design. The two moral transgression vignettes were taken from other research [23], specifically the bribe-accepting congressman and the shoplifter; chosen because they were judged to be harshest by control participants in previous research [9]. Moral virtue vignettes were written in a similar style to describe two altruistic acts (a generous gift to a homeless family and a Good Samaritan preventing a mugging). The two control vignettes depicted non-moral events (a student choosing a major and a waiter interacting with co-workers).

The order of vignettes in each condition was counterbalanced and followed by a 14-cm line ranging from *extremely morally bad* to *extremely morally good* to rate the morality of each situation. Participants were asked to make a slash at the point on the continuum corresponding to their impressions. These marks were then converted to scores ranging from 0 to 100, with lower scores indicating harsher moral judgments.

#### Taste perception

Following the moral judgment task, participants began part two of the experiment. Everyone was given the same beverage to taste (diluted blue Gatorade, 1 part Gatorade to 10 parts water), which was chosen because it is not particularly sweet, bitter, or domineering in any other taste. They were not told the identity of the beverage, but were shown the ingredients to review for potential allergies and post-experimental interviews indicated that they could not identify the beverage. Beverages were administered in a single one-teaspoon (4.93-mL) dose in a small cup. Participants were instructed to drink each dose in its entirety in a single swift motion, “as if they were drinking a shot.” They rated the beverage on another 14-cm line representing a continuum from *very disgusting* to *very delicious*. Participants were asked to make a slash at the point on the continuum corresponding to their impressions While it is a possible measurement limitation that the moral judgment and taste perception scales were both anchored with positive judgments on the right and negative judgments on the left, this would be unlikely to have a significant effect on the outcome of this study. Further, it would call into question the majority of survey/self-report data conducted in the social/behavioral sciences. However, it is worth noting that the scales should ideally be counterbalanced across and within participants, and future research should consider such an approach. These marks were then converted to scores ranging from 0 to 100, with lower scores indicating stronger perceptual disgust. The beverage was also rated in terms of its perceived sweetness and bitterness using a 7-point scale ranging from *not at all* (1) to *very much* (7).

#### Demographics

Participants were also asked to provide some basic demographic information. They also rated how sweet, bitter, neutral, and disgusting they found their beverage using a 7-point scale ranging from *not at all* (1) to *very much* (7). Finally, they were asked to write down what they thought the study was about.

## Results

Since none of the participants correctly guessed the hypothesis, all 60 were used in the primary analyses. Table 1 confirms that participants actually perceived the moral transgression vignettes as morally wrong, the virtuous events as morally right, and the control vignettes as morally neutral, with higher numbers indicating morally right events and lower numbers indicating morally wrong events.

**Table 1:** Participant ratings of moral vignettes.

Vignette Type	Transgression	Control	Virtue
	17.00 (11.21)	56.08 (19.01)	88.90 (10.83)

Note. Mean ratings of vignettes with standard deviations in parentheses. The higher or lower the numbers, the more the events were judged to be morally right or wrong, respectively.

**Figure 1:**
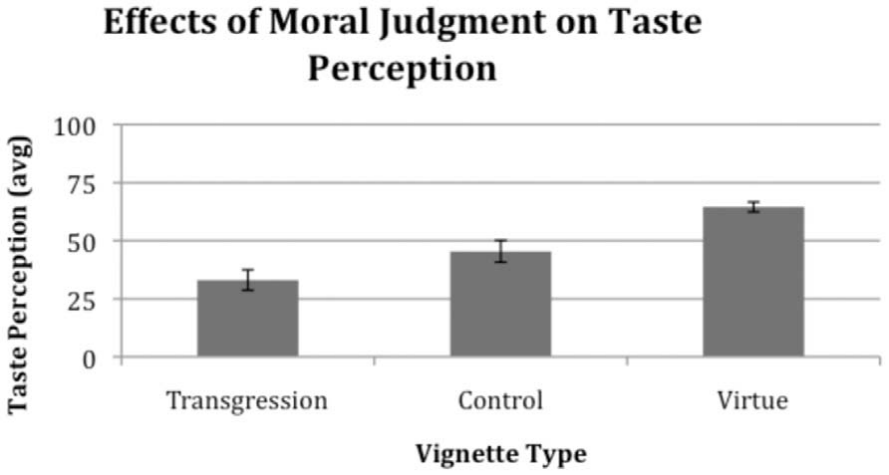
Participants’ mean taste perceptions as a function of moral judgments, with higher numbers indicating more delicious taste perceptions.

To determine the effects of the moral vignette manipulation, a one-way ANOVA of taste perception was conducted. Results revealed a significant effect of vignette type, *F*(2, 57)  = 16.43, *p*<.001, η_p_^2^ = .366. Planned contrasts on the data in Figure 1 showed that participants perceived the beverage to be significantly more disgusting in the transgression condition (*M* = 33.10, *SD* = 19.72, *n* = 20) than in the control condition (*M* = 45.40, *SD* = 21.05, *n* = 20), *t*(57) = 2.221, *p*s = .030, *d* = −0.603. The other contrast revealed that participants perceived the beverage to be more delicious in the virtue condition (*M* = 64.60, *SD* = 9.41, *n* = 20), than in the control condition, *t*(57) = 3.466, *p* = .001, *d* = 1.261. A post-hoc Tukey test confirmed that the transgression and virtue conditions yielded significantly different taste perceptions, *p*<.001. In addition to rating how disgusting/delicious the target beverage was, participants also rated how sweet and bitter they perceived it to be. To determine whether vignette type affected participants’ sweetness and bitterness ratings of the target beverage, a MANOVA was conducted on *both* sweetness and bitterness ratings (with vignette type as the between-person factor) and revealed no significant differences in either the sweetness or bitterness ratings as a function of vignette type, *F*s <1. This finding suggests that disgust, not bitterness per se, is related to moral transgressions.

A regression was also performed to test whether taste perception could be predicted by moral judgments. Results showed that 46.2% of the variance in taste perception was accounted for by participants’ moral judgments, *t*(58) = 7.063, *p*<.001, β = .680. Together, these results confirm the primary hypothesis that abstract moral processing can influence embodied gustatory experiences in both directions (disgust and delight).

## Discussion

The purpose of the present experiment was to investigate the strength of the connection between moral processing and embodied experience by determining whether the direction of previous research’s primary finding could be reversed [9]. Participants were presented with moral transgressions, virtues, or control events prior to rating the same target beverage in terms of how disgusting, delicious, bitter, and sweet they found it. We hypothesized that that those exposed to moral transgressions, virtues, or control events would perceive the beverage to be more disgusting, delicious, or neutral tasting, respectively. Results showed that exposure to different moral (or non-moral) events did indeed elicit the predicted taste perceptions, thereby confirming the bidirectional relationship between morality and embodied disgust. These findings not only bolster the link between moral transgressions and embodied states of disgust, but also show that the opposite is true–moral virtue is connected to embodied gustatory delight, which appears to be a novel finding in morality research. Hence, the current study both replicates and extends the findings reported elsewhere [9].

Other researchers recently conducted a clever experiment that resulted in a similar effect [21]. Specifically, one study showed that Christians were significantly more likely to rate a neutral tasting beverage (a solution of lemon water) as disgusting after copying a passage from Richard Dawkins’ *The God Delusion* or the Qur’an compared to a control text. However, in another study, they removed the effect by directing participants who processed rejected religious beliefs to subsequently wash their hands, thereby corroborating the moral purity metaphor advanced in other research [14], [5], [6]. Although this research presents an exciting finding, it did not involve explicit measures of moral disgust. Truly assessing the bidirectionality of an effect requires the use of very similar methodologies and measures, like those reported here and elsewhere [9]. Using converging methodologies enables us to further specify the relationship between physical source domains (e.g., taste) and abstract target domains like morality from conceptual metaphor theory, with respect to how such embodied and abstract domains are ultimately represented and processed.

The present research also suggests that interoceptive perceptions (i.e., emotional states and affective information) are fundamental to how we represent and process morality. This line of reasoning has been described by others [24], and is typically referred to as *social intuitionism* [1], [10], [25]. This two-stage theory predicts that emotional states influence moral judgments in a direct and automatic manner, whereas reason-driven states are supported by controlled processes, which are not always required, motivated, or both. Other programs of research support such a dual-process model of judgment. For example, researchers have found that negative emotional arousal (induced sadness) resulted in lower acceptance of unethical behavior in the Ultimatum Game [26], and others found a similar finding for induced disgust [27]. Together, such results suggest that emotions play a crucial role in decision making processes, providing support for social intuitionist approaches to morality.

One could argue that the present disgust-embodiment results could be explained simply as negative arousal effects on judgment. This is indeed a valid possibility and a limitation of the present research is that it cannot disentangle embodied disgust from more general negative emotional arousal. Research on the modality-specific effects of embodied disgust and moral transgressions favor the embodiment approach [14], but no research to date has explicitly contrasted the effects of general arousal and embodied arousal on moral judgments/decision making. This represents an important direction for future studies. However, an alternative possibility is that emotional and embodied arousal are essentially the same and difficult to disentangle. According to the influential *somatic marker hypothesis*, emotions refer to a unique family of representations that carry information about various homeostatic changes *in the body* across a broad array of contexts, with contextual features represented in the form of external stimuli with corresponding response options [28]. This embodied, emotional information is encoded and stored so that similar subsequent situations and decision-inducing contexts will re-activate one’s “somatic markers” to facilitate decision making. Simply put, *all* emotional information seems to be fundamentally *embodied* at its core. An important consideration would be to determine the implicit and explicit effects of embodied and emotional information, as well as the conditions under which they can be made salient/conscious (and potentially overridden).

Aside from these limitations, the present research provides additional evidence that sensoriperceptual and emotional states share the same conceptual space as morality, although the precise underlying representation(s) of morality remain unclear since the focus of the current research was on moral processing rather than representational structure. Nevertheless, this is the first empirical demonstration to (1) explicitly assess physical and moral disgust and delight and (2) reveal the bidirectional effects of physical and moral disgust. Future research should try to further study and understand the representation of morality, in addition to specifying the time-course of the embodied effects reported here and elsewhere, to determine the extent to which they influence moral thoughts and actions (i.e., prosocial behavior) in a broader ecological sense.

The present research is part of a substantial and growing number of studies providing further evidence that abstract conceptual representations are grounded in embodied information. Indeed, recent research in other conceptually abstract domains like aesthetics has also revealed that affective perceptual states can influence aesthetic judgments [29], and evidence from cognitive neuroscience suggests that evolutionarily older brain regions (i.e., limbic system) are implicated in higher-order cognition and judgment making [30], [28]. This work thus provides a framework for understanding how traditionally described metaphorically-based mechanisms [17], [18] could operate in the representation and processing of abstract concepts.
